# High Circulating Levels of ANGPTL2: Beyond a Clinical Marker of Systemic Inflammation

**DOI:** 10.1155/2017/1096385

**Published:** 2017-08-24

**Authors:** Nathalie Thorin-Trescases, Eric Thorin

**Affiliations:** ^1^Faculty of Medicine, Montreal Heart Institute, Montreal, QC, Canada; ^2^Department of Surgery, Montreal University, Montreal, QC, Canada

## Abstract

Angiopoietin-like 2 (ANGPTL2) is a proinflammatory protein belonging to the angiopoietin-like family. ANGPTL2 is secreted and detected in the systemic circulation. Different observational clinical studies reported that circulating levels of ANGPTL2 increase significantly in various chronic inflammatory diseases and showed associations between ANGPTL2 levels and diagnosis and/or prognosis of cardiovascular diseases, diabetes, chronic kidney disease, and various types of cancers. However, these studies did not address the following questions: (a) what are the sources of circulating ANGPTL2? (b) How and by which mechanisms an increase in circulating ANGPTL2 contributes to the pathogenesis of chronic inflammatory diseases? (c) Does an increase in circulating levels of ANGPTL2 measured in a well-defined chronic medical condition originate from a specific cell type? Mechanistic hypotheses have been proposed based on studies performed in mice and cultured cells, and proinflammatory, prooxidative, proangiogenic, proliferative, and antiapoptotic properties of ANGPTL2 have been reported. The aim of this review is to propose answers concerning the potential sources of circulating ANGPTL2 and its common pathological properties associated with various chronic inflammatory diseases and death in humans. We believe that high circulating ANGPTL2 levels are more than an inflammatory marker and may reflect the senescent cellular load of an individual.

## 1. Introduction

Angiopoietin-like 2 (ANGPTL2) belongs to the angiopoietin-like family, a family of eight (ANGPTL1–8) members of glycoproteins [1]. Their structure is similar to that of angiopoietins, and they possess a typical N-terminal coiled-coil domain, a short linker peptide, and a C-terminal fibrinogen-like domain [2]. ANGPTL2 was cloned, expressed, and characterized for the first time in 1999; it is a glycosylated protein of 493 amino acids, of 57 kDa (64 kDa with the glycosylations) [2]. One report proposed that ANGPTL2 could be cleaved into domain fragments by the tolloid-like 1 protease and be inactivated, at least *in vitro* in cultured cancer cells [3]. It is expressed in many tissues and is secreted in the systemic circulation [2, 4, 5]. Circulating levels of ANGPTL2 in human healthy volunteers range from ~1 to 3 ng/ml [6] (Tables 1, 2, and 3), with similar levels between males and females [5, 7–9]. Higher values are measured in patients with chronic or autoimmune diseases, and discrepancies between circulating levels reported in different studies are most likely due to the differences in age, ethnic background, geographical location, clinical characteristics, and/or severity of the disease and to a lesser extent to the ELISA kit used to quantify ANGPTL2 levels (Tables 1, 2, and 3).

ANGPTL2 is a multifaceted protein, displaying both physiological and pathological functions (for reviews, [6, 10]). ANGPTL2 was originally acknowledged for its proangiogenic [2, 11, 12] and antiapoptotic capacities [13]. More recently, beneficial angiogenic properties of ANGPTL2 were reported in the context of stroke [14] and one study demonstrated that ANGPTL2 displays antithrombotic properties [15]. ANGPTL2 may also contribute to vasculogenesis [16]. An important physiological property of ANGPTL2 is that it increases survival and expansion of hematopoietic stem and progenitor cells [17–22]. Recently, it has been reported that ANGPTL2 may also be a key player in intestinal stem cells by regulating intestinal epithelial regeneration [23]. ANGPTL2 may maintain tissue homeostasis by promoting adaptive inflammation and tissue reconstruction [10], and it also protects against lung fibrosis [24] and promotes beneficial innate immune response [25]. Nonetheless, circulating ANGPTL2 levels increase gradually with age in the general population [26].

ANGPTL2 is better acknowledged for its association with multiple chronic diseases, in particular in various types of cancers [3, 27–43]. High circulating levels of ANGPTL2 have indeed been proposed to be a biomarker for early diagnosis and recurrence of various types of cancers (Table 2). Furthermore, increased levels of ANGPTL2 were reported in diabetes [5, 44–47], chronic kidney disease [9, 46, 48, 49], cardiovascular diseases [5, 7, 8, 26, 50–57], metabolic disorders including obesity [5, 55, 58–60], and other diseases (for review, [6]), including autoimmune diseases such as dermatomyositis [61] and rheumatoid arthritis [62] (Tables 1 and 3). ANGPTL2 levels in the circulation were also reported to be a predictor of major adverse cardiac events and death in diabetic [45] and renal transplant [48] patients. Despite this multitude of evidence of pathological higher circulating levels of ANGPTL2 in different diseases, it remains to determine (a) what are the sources of circulating ANGPTL2? (b) How and by which mechanisms an increase in circulating ANGPTL2 contributes to the pathogenesis of chronic inflammatory diseases? (c) Does an increase in circulating levels of ANGPTL2 measured in a well-defined chronic medical condition originate from a specific cell type?

## 2. Putative Receptors of ANGPTL2

Once ANGPTL2 is secreted in the circulation, it forms multimers and exerts its cellular effects, locally or distantly, depending on the local or distant expression of ANGPTL2 receptors or binding proteins on target cells. Until recently, ANGPTL proteins were considered as orphan ligands, but some candidates have been proposed: the team of Oike and Tabata first hypothesized that Toll-like receptor 4 (TLR4) could be potential receptors for ANGPTL2 in endothelial cells and monocytes, since ANGPTL2 possesses a fibrinogen-like domain and that fibrinogen acts as an intrinsic TLR4 ligand [55]. The same team then demonstrated that integrin *α*5*β*1 binds ANGPTL2 in adipocytes, endothelial cells, and cancer cells because the effects of recombinant ANGPTL2 protein were blocked by integrin *α*5*β*1-neutralizing antibodies [5, 26, 63]. On the other hand, immune inhibitory receptor human leucocyte immunoglobulin-like receptor B2 (LILRB2) and its mouse orthologue-paired immunoglobulin-like receptor (PIRB) have been reported to bind several ANGPTL proteins, especially ANGPTL2 and ANGPTL5, in hematopoietic cells (HSC) [22]. The binding of ANGPTL2 to LILRB2 might be restricted to HSC [22], platelets [15], and some pancreatic cancer cells [29]. Finally, Guo et al. demonstrated that intracellular ANGPTL2 binds to the intracellular C-terminal domain of AT1A receptors in HEK-293 cells and kidney cells and that it specifically promotes AT1A recycling to the plasma membrane, with concomitant recovery of AT1A receptor signal functions [64, 65]. Hence, depending on the cell type, different receptors for ANGPTL2 have been reported. Whether ANGPTL2 signaling depends on the type of target cells expressing integrin *α*5*β*1 or LILRB2 receptors (or additional unidentified receptors or binding proteins) is not clearly known.

## 3. Sources of Circulating ANGPTL2

### 3.1. Adipocytes

ANGPTL2 is abundantly expressed in white adipose tissue in mice, especially visceral adipose tissue, and circulating ANGPTL2 correlates with adiposity in humans [5]. Circulating ANGPTL2 levels are positively correlated with BMI, weight, visceral fat surface, and other anthropometric parameters in the general population [9, 51], in overweight [59] and in obese [5] and severely obese [60] subjects. Visceral fat is therefore believed to be the greatest source of circulating ANGPTL2. Consequently, ANGPTL2 has been categorized as an adipokine [66], a protein secreted by adipocytes, and it has been proposed to counter-interact the beneficial effects of adiponectin [67]. Accordingly, the reduction in circulating ANGPTL2 levels measured after a bariatric surgery in severe obese patients were correlated with a reduction in leptin levels and inversely correlated with an increase in adiponectin levels [60]. In addition, we observed that in patients with cardiovascular risk factors, including a high BMI and exhibiting elevated circulating ANGPTL2 levels, the slimmer the patient, the lower his/her ANGPTL2 concentration [7]. After 3 months of diet and physical training, ANGPTL2 levels measured in overweight healthy subjects reflected weight reduction and improvement in metabolic parameters [7]. Hence, these data would suggest that adiposity is the driver of ANGPTL2 production.

But it is not so clear-cut. We reported that in overweight patients with acute coronary syndrome, 3 months of physical training reduced significantly plasma ANGPTL2 levels independently of a weight loss: while ANGPTL2 levels were reduced by 26% in men with acute coronary syndrome, body mass, lean and fat mass, waist circumference, and BMI were not affected by the exercise training program [8]. On the other hand, we recently reported that in severely obese patients, the relatively low reduction of ANGPTL2 levels after bariatric surgery (0% at 6 months, 18% at 1 year) does not reflect the drastic weight reduction (27% at 6 months, 37% at 1 year), but rather improvement in diabetes, dyslipidemia, and inflammation [60]. The fact that changes in ANGPTL2 were not associated with body weight loss or fat mass reduction suggests that reduction of ANGPTL2 levels after bariatric surgery or physical training is not simply a marker of adiposity. This also suggests that ANGPTL2 may be produced and secreted by different cell types, not only adipocytes and/or that despite significant weight loss and reduction in visceral adipose tissue mass, damaged adipocytes still produce ANGPTL2. In the case of severely obese patients, it is likely that dysfunctional and hypertrophied adipocytes are still present after bariatric surgery and are slow to eliminate; these dysfunctional and hypertrophied adipocytes could still produce ANGPTL2 and other inflammatory cytokines, which could further promote ANGPTL2 expression, in a vicious circle.

### 3.2. Heart-Derived ANGPTL2

ANGPTL2 is abundantly expressed in the heart, and its cDNA was originally isolated from human and mouse hearts [2]. Higher circulating ANGPTL2 levels were reported in heart failure patients, and higher levels were associated with increased (~3-fold) risk of heart failure [52]. Although adipose tissue was assumed to be the main source of ANGPTL2 in the latter study, the authors questioned whether ANGPTL2 derived from cardiac cells could also contribute to cardiac dysfunction observed in heart failure patients [52]. Recently, it has been demonstrated that the heart can produce and secrete ANGPTL2: in patients with dilated cardiomyopathy, a difference between blood ANGPTL2 concentration measured in the coronary sinus and that measured in the aortic root reflected ANGPTL2 secretion directly from the heart, including from cardiomyocytes [68]. In addition, the authors showed that heart-derived ANGPTL2 contributed to accelerate heart failure, by lowering ventricular contractility and by decreasing myocardial energy metabolism [68]. Interestingly, this study showed that in mice, after targeted suppression of cardiac ANGPTL2 production by treatment of the mice with AAV6-shAngptl2, circulating ANGPTL2 levels were significantly reduced and this was associated with a better cardiac function and metabolism [68], confirming that ANGPTL2 in the circulation may origin, at least partly, from the heart. Nevertheless, studies on heart-derived ANGPTL2 and on its role in the cardiac function remain very sparse.

### 3.3. Kidney-Derived ANGPTL2

The first link between ANGPTL2 and kidney disease has been described in patients with diabetic glomerulopathy in whom upregulation of ANGPTL2 expression in microvascular lesions was associated with a higher prevalence of renal insufficiency [69]. Few years later, in a general Japanese population, elevated circulating ANGPTL2 levels mainly related to albuminuria were found to be an independent predictor of chronic kidney disease prevalence [9]. Similarly, elevated circulating ANGPTL2 levels in association with albuminuria were reported in patients with diabetic nephropathy [46]. Although these studies suggest a role of ANGPTL2 in renal dysfunction, they did not identify the source of ANGPTL2. Could the kidney itself secrete ANGPTL2? This was proposed by Morinaga et al. in a mouse model of renal fibrosis in which high levels of circulating ANGPTL2 levels may originate from damaged kidneys [49, 70]. We recently reported that in patients with severe chronic kidney disease (stage 5), kidney transplantation dramatically reduced serum ANGPTL2 levels, raising the possibility that the diseased kidney was a source of ANGPTL2 [48]. Another possibility is that after kidney transplant, clearance of ANGPTL2 is improved and thus circulating levels of ANGPTL2 reduced, whatever the source of the protein. However, ANGPTL2 is a large protein, which potentially forms multimers in the circulation [9] making its glomerular excretion and its renal clearance unlikely. This has never been confirmed for ANGPTL2, but angiopoeitin 2, a glycoprotein with similar structure and molecular weight, is not excreted, not detectable in urine, and not eliminated by dialysis [71, 72]. Thus, the fact that ANGPTL2 levels are significantly reduced after kidney transplantation strongly suggests that the kidney is a significant source of ANGPTL2 [48]. In addition, ANGPTL2 plays an active role in kidney disease: indeed, we observed that 3 months after renal transplantation, ANGPTL2 circulating levels were associated with aortic stiffness, central pulse pressure, and renal dysfunction, and the risk of mortality was significantly higher (3.9-fold) in patients with the highest posttransplant ANGPTL2 circulating levels [48]. Altogether, these studies suggest that the kidney could be a source of ANGPTL2 and that it plays an active role in kidney disease.

### 3.4. Other Sources of Circulating ANGPTL2

Because ANGPTL2 is proinflammatory, an alternative source of circulating ANGPTL2 is the macrophages, either derived from bone marrow [73] or infiltrating macrophages [26, 56, 74]. In our hands, human leukocytes did not produce detectable levels of *ANGPTL2* mRNA [54], but immune cells have been reported to express it [5, 56, 73]. Immune cells are likely not the main contributor to the circulating pool of ANGPTL2, but endothelial cells (from heart, kidney, and adipose tissue) could be an interesting alternative. We reported that senescent endothelial cells from atherosclerotic patients express high levels of *ANGPTL2* mRNA [50, 75]. We also demonstrated that endothelial cells, but not vascular smooth muscle cells, produced ANGPTL2 and that ANGPTL2 was abundantly expressed in endothelial cells and macrophages in the atherosclerotic mouse aorta [50]. Circulating levels of ANGPTL2 are associated with atherosclerotic lesions in mice [50] and in humans [26]. In addition, ANGPTL2 causes endothelial dysfunction [26, 76]. Thus, endothelial cell-derived ANGPTL2 could be a source of circulating ANGPTL2. In accordance with this hypothesis, we recently reported that in patients with severe chronic kidney disease, high ANGPTL2 circulating levels measured after kidney transplantation were associated with high serum endothelin-1 levels [48]. In addition, we showed that in patients with acute coronary syndrome, the reduction of ANGPTL2 levels induced by the training program correlated with endothelial function measured at baseline: better initial endothelial function correlated with lower ANGPTL2 levels reached after exercise [8]. Dysfunctional (mouse, human) and/or senescent (human) endothelial cells could therefore produce ANGPTL2, contributing to the proinflammatory environment.

Elevated serum ANGPTL2 levels have been reported in various types of cancer, making ANGPTL2 a potential biomarker for early diagnosis, prognosis, and recurrence of cancer (Table 2). Cancer cells produce and secrete ANGPTL2; this was observed in breast cancer cells [32] and in esophageal cancer cell lines [34]. In addition, it has been proposed that primary tumour might be the source of elevated serum ANGPTL2 measured in colorectal cancer patients, since high levels of ANGPTL2 in tumour tissues and in matched serum samples were both associated with tumour size, distant metastasis, and cancer stage [40]. Similarly, the same team proposed that elevated ANGPTL2 circulating levels measured in gastric cancer patients are likely produced by the tumour and the adjacent normal mucosa [39].

In summary, ANGPTL2 in the circulation mainly comes from the visceral adipose tissue, with a contribution of adipocytes, endothelial cells, and infiltrated macrophages, particularly in obese patients in whom augmented fat tissue mass associates with a matched microcirculation density and infiltrated inflammatory cells. In addition, ANGPTL2 derived from cardiac and kidney cells also likely constitutes another pool of circulating ANGPTL2. Finally, in the context of cancer, the tumours may also be the source of elevated ANGPTL2 systemic expression.

## 4. How an Increase in Circulating ANGPTL2 Contributes to the Pathogenesis of Chronic Inflammatory Diseases?

### 4.1. ANGPTL2-Induced Inflammation

The obvious link between an increase in circulating levels of ANGPTL2 and chronic diseases such as cancer and cardiovascular diseases is systemic inflammation. Both cancer and cardiovascular diseases are associated with chronic low-grade inflammation, in which sustained overproduction of proinflammatory ANGPTL2 could contribute [55].

In the context of diabetes and obesity, adipocytes, endothelial cells, and infiltrated macrophages produce ANGPTL2 leading to an inflammatory response mediated through activation of the NF*κ*B pathway: ANGPTL2 binds to integrin *α*5*β*1, activates Rac1, and thus translocates NF*κ*B to the nucleus by increasing the degradation of its inhibitor I*κ*B, resulting in NF*κ*B-dependent inflammatory gene expression [5]. This inflammation, if chronic, extends to the pancreas where insulin secretion defects occur, to the skeletal muscle in association with insulin resistance and to the liver, where both insulin resistance and hyperglycemia develop. Thus, adipocyte-derived inflammatory ANGPTL2 was proposed to link obesity to insulin resistance [5]. Accordingly, beneficial antidiabetic treatment with pioglitazone reduced circulating ANGPTL2 levels [5]. In the context of atherosclerosis in mice, recombinant ANGPTL2 protein promotes inflammation of endothelial cells, the expression of proinflammatory cytokines such as IL6 and TNF*α* and adhesion molecules such as ICAM1 and P-selectin, and accelerates the formation of the atherosclerotic plaque [50]. On the other hand, endothelial dysfunction, macrophage infiltration/activation, and perivascular adipose tissue cooperatively contribute to increase ANGPTL2 production, leading to NF*κ*B-dependent chronic inflammation in the vessel wall [26]. To further strengthen the inflammatory role of ANGPTL2, circulating ANGPTL2 levels are closely associated with inflammatory markers such as CRP in obesity [5], diabetes [53], acute coronary syndrome [57], heart failure [52], cancer [41] and in the general population [9, 51]. Circulating levels of ANGPTL2 are also positively correlated with levels of the proinflammatory cytokine TNF*α* or its receptor TNFR1 in diabetes [45], obesity [60], and heart failure [52]. In the context of cancer, ANGPTL2-induced inflammation creates a deleterious environment that favours genomic instability and DNA damage and thus contributes to all stages of tumour development, from initiation to progression [27, 28, 32, 34, 40]. On the other hand, the tumour microenvironment activates transcription factors such as nuclear factor of activated T-cells (NFAT), c-Jun, and activated transcription factor 2 (ATF2), leading to an increased transcriptional expression of ANGPTL2 [31]. In cancer cells, ANGPTL2 promotes epithelial to mesenchymal transition, a critical step for tumour invasive properties and metastasis, via activation of the TGF*β*-Smad pathway [27]. ANGPTL2 also promotes extracellular matrix degradation via activation of matrix metalloproteinase MMP-9 under the control of p38 MAPK through integrin *α*5*β*1 receptors [3]. In addition, ANGPTL2 expression being sensitive to hypoxia [11], epigenetic DNA methylation of *ANGPTL2* promoter occurs in hypoxic tumour cells, leading to an increase in ANGPTL2 expression ultimately reinforcing the inflammatory response and the aggressive cancer cell phenotype [3]. It has been proposed that ANGPTL2 secreted from cancer cells activates the integrin *α*5*β*1/Rac1 pathway, promoting both autocrine and paracrine effects, increasing tumour cell invasion, motility, tumour angiogenesis, and thus tumour metastasis [31].

### 4.2. ANGPTL2-Induced Noninflammatory Responses

Despite its potent proinflammatory properties, ANGPTL2 also exerts noninflammatory responses in various target organs. In the heart, for example, it has been recently demonstrated that ANGPTL2 reduces ventricular contractility by downregulating AKT-SERCA2A signalling, affecting cardiac calcium handling, leading to cardiac dysfunction independently of an inflammatory response [68]. In addition, ANGPTL2 also reduced cardiac energy metabolism and the authors proposed that cardiac ANGPTL2 expression was regulated, at least partially, by myocardial O_2_ levels: physical training-induced physiological remodelling was associated with a decrease in cardiac expression of ANGPTL2, while ANGPTL2 expression increased in a pathological myocardial hypoxia-associated remodelling [68]. Interestingly, we observed that circulating levels of ANGPTL2 were associated with the cardiopulmonary function of patients with cardiovascular diseases: the better the VO_2_ max, the lower baseline circulating levels of ANGPTL2 [7].

Another target organ in which ANGPTL2 may exert responses other than inflammation is the kidney. ANGPTL2 is a proangiogenic factor, and this may explain the abnormal vessel growth observed in diabetic nephropathy [46]. More importantly, ANGPTL2 promotes renal fibrosis by activating the expression of the growth factor TGF*β*1 through *α*5*β*1 integrin-mediated activation of extracellular signal-regulated kinase [49]. In addition, TGF*β*1 upregulates ANGPTL2 expression via the inhibition of miR-221 that negatively controls ANGPTL2 [49]. Thus, in combination with inflammation, renal fibrosis induced by ANGPTL2 contributes to chronic kidney disease. This fibrotic property of ANGPTL2 may also explain the association between elevated circulating ANGPTL2 levels and aortic stiffness (assessed by carotid-femoral pulse wave velocity) observed in our recent study in patients with very severe chronic kidney disease [48]: by promoting fibrosis, ANGPTL2 may contribute to large artery stiffening. Interestingly, in this latter study, we observed no significant association between ANGPTL2 levels and inflammation as measured by multiple circulating cytokine levels, suggesting that the deleterious effects of ANGPTL2 on renal function were independent of these inflammatory mediators [48].

Another property of ANGPTL2 is its prooxidant capacity. ANGPTL2 derived from inflammatory cells such as activated macrophages and neutrophils, and ANGPTL2-induced activation of NF*κ*B-dependent pathway could be sources of reactive oxygen species (ROS) leading to oxidative stress [28]. Furthermore, ROS activate the phosphorylation of the transcription factors c-Jun and ATF2, thereby increasing the expression of their target genes, including ANGPTL2 itself [27, 28]. This effect was mostly described in cancer cells [27, 28] but is likely to occur in other cell types, for example, in the vascular wall. Accordingly, we reported that ANGPTL2-induced vascular endothelial dysfunction in mice could be partially reversed by the antioxidant N-acetyl cysteine [76]. In addition, atorvastatin (with anti-inflammatory and antioxidant properties) slightly reduced serum ANGPTL2 and improved left ventricular function in patients with acute myocardial infarction [77].

ANGPTL2 displays other deleterious properties, such as activation of metalloproteinases, leading to extracellular matrix degradation that could contribute to abdominal aortic aneurysm and deleterious arterial remodelling [56]. Activation of metalloproteinases by ANGPTL2 via activation of p38 MAPK was also reported in cancer cells [3], contributing to increase tumour cell metastasis.

Finally, another property of ANGPTL2 indirectly related to inflammation is its potential capacity to contribute to cellular senescence. Cellular senescence is associated with aging and various chronic diseases, and we originally isolated ANGPTL2 from senescent vascular endothelial cells of patients with severe coronary artery disease [75]. Oxidative stress-induced premature senescence observed in endothelial cells from active smokers with severe coronary artery disease was associated with higher (4-folds) levels of *ANGPTL2* mRNA [75]. After chronic treatment of these cells with the antioxidant N-acetyl cysteine [50], or in cells isolated from past smokers [75], the expression of *ANGPTL2* mRNA was reduced and senescence delayed. Similarly, among other genes related to vascular remodelling and inflammation, higher expression of *ANGPTL2* mRNA has been recently reported in senescent platelet [78]. Thus, the intriguing possibility arises that ANGPTL2 could be produced by senescent cells, including senescent adipocytes, cardiac, and renal cells, and/or contributes to the maintenance of senescence. ANGPTL2 has been shown to contribute to the senescence-associated secretory phenotype (SASP) in aged induced pluripotent stem cells from patients with Werner syndrome [79], a progeria leading to premature aging, increased fat mass, and cardiovascular diseases [80]. ANGPTL2 was also listed as a SASP molecule in senescent fibroblasts [81]. Whether ANGPTL2 contributes to SASP in senescent adipocytes, endothelial, cardiac, and renal cells remains to be demonstrated, but we hypothesize that the high circulating ANGPTL2 levels observed in patients with chronic inflammatory diseases could reflect the senescent cellular load of an individual. Indeed, accumulation of senescent cells might be a source of increased circulating SASP-derived inflammatory molecules [82], such as increased systemic levels of ANGPTL2. The molecular mechanisms underlying SASP-derived ANGPTL2 secretion and ANGPTL2-induced senescence are unknown.

In summary, it is very likely that ANGPTL2-induced oxidative stress, fibrosis, tissue remodelling, and cellular senescence, in association or not with inflammation, all contribute and synergize to promote the pathogenesis and progression of chronic diseases such as cancer and cardiovascular diseases. ANGPTL2 activates molecular pathways where the integrin *α*5*β*1/Rac1/NF*κ*B-dependent pathway seems to play a key role in a multitude of cell types and pathologies. Regarding the molecular basis of ANGPTL2, more complete information can be found in other reviews [6, 10].

Altogether, these multiple properties of ANGPTL2 raise the following questions: does circulating ANGPTL2 play any roles in the local cellular microenviroment, and if so, how? The team of Zhang and Zheng, known for their discoveries on the role of ANGPTL2 in hematopoietic cell proliferation [21] and ANGPTL2 receptor LILRB2 [22], recently elegantly demonstrated that secretory proteins produced by HSC, such as ANGPTL2, ANGPTL3, and thrombopoietin, undergo exosomal maturation and release that is controlled by a vacuolar sorting protein VPS33B [83]. Exosomal process, that is, endosome formation, their loading and trafficking within the cell toward the plasma membrane, their fusion with the plasma membrane, and their release to the extracellular space, is a complex process that occurs upon a microenvironment pressure selection or stimulation [84]. Concerning ANGPTL2, Gu et al. demonstrated that within HSC, ANGPTL2 is primarily located in multivesicular bodies and that VPS33B is crucial to regulate the release of ANGPTL2 that ultimately will maintain stemness [83]. They therefore suggested that ANGPTL2 exerts autocrine local effects in the regulation of HSC stemness [83]. However, this autocrine effect may be specific to HSC and not be present in other cell types, such as endothelial cells, since deletion of *vps33b* in endothelial cells did not affect the expression levels of ANGPTL2 and since VPS33B knock-out in HUVEC did not affect exosome maturation and release of ANGPTL2 [83]. Interestingly, minimal *LILRB2* mRNA expression was observed in human aortic endothelial cells, in contrast to robust integrin *α*5*β*1 expression in endothelial cells [26]. Altogether, these data suggest that ANGPTL2 secreted by HSC in the circulation may exert local roles in its microenvironment. In pancreatic cancer cell lines, an autocrine loop between LILRB2 and its ligand ANGPTL2 has also been demonstrated, leading to early stages of cancer [29], suggesting again a local action of ANGPTL2. Whether this is true for all cell types remains to be determined, since depending of the cell type, different receptors for ANGPTL2 have been reported.

## 5. Does an Increase in Circulating Levels of ANGPTL2 Measured in a Well-Defined Chronic Medical Condition Originate from a Specific Cell Type?

**Figure 1:**
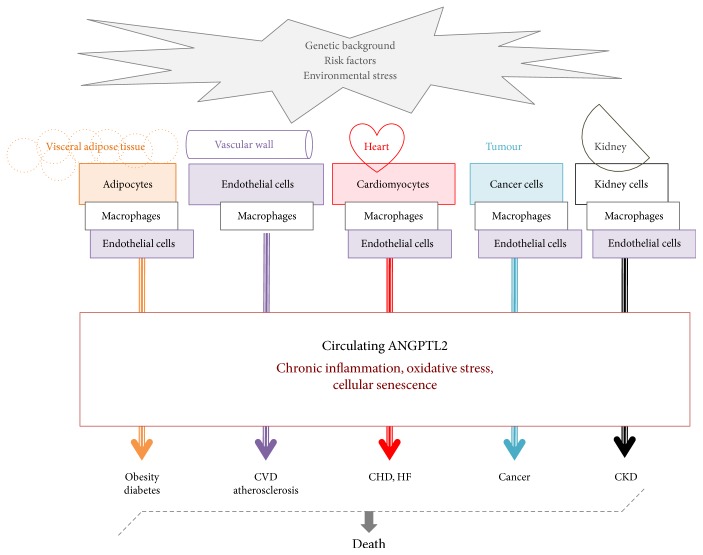
Hypothetic schematic representation of the pathological origins and effects of high circulating ANGPTL2 levels. ANGPTL2 derived from activated adipocytes, macrophages, endothelial cells, cardiomyocytes, cancer cells, or kidney cells increase circulating ANGPTL2 levels. Through its autocrine and paracrine proinflammatory and prooxidative properties, ANGPTL2 contributes to maintain a chronic low-grade systemic inflammation and induces cellular senescence, favouring the pathogenesis of various diseases, leading ultimately to death. It is likely that ANGPTL2 derived from different cell types cooperates and synergizes to favour the pathogenesis of one chronic disease, but one cell type may also be predominantly implicated. CHD: coronary heart disease; CKD: chronic kidney disease; CVD: cardiovascular disease; HF: heart failure.

It is very unlikely that elevated circulating levels of ANGPTL2 are a biomarker of a specific disease. ANGPTL2 circulating levels cannot be associated with a specific disorder such as cancer, even not with a specific type of cancer: high ANGPTL2 concentrations were reported to be a potential marker of breast [32], esophageal [34], gastric [39, 42], and colorectal [38, 41] cancers (Table 2), but they are rather a marker of cellular dysfunction and associate with systemic inflammation, hypoxia, and oxidative stress common to the microenvironment of cancer cells [40]. If high circulating levels of ANGPTL2 do not reflect a specific disorder, they are an indicative of a deleterious and pathological health status, reflecting dysfunction of adipose tissue, heart, and/or kidney cells, indicating the development and progression of a chronic disease or predicting major adverse cardiac events and death [45, 48]. Elevated circulating levels of ANGPTL2 thus reflect a cellular dysfunction pathway that is common to different chronic diseases. However, it is possible that higher circulating levels of ANGPTL2 observed in a particular disease reflect dysfunction of a specific cell type: ANGPTL2 derived from activated cancer cells may increase circulating ANGPTL2 levels, contributing to the pathogenesis of cancer. It is likely that ANGPTL2 derived from different cell types cooperates and synergizes to favour the pathogenesis of one chronic disease, but one cell type may also be predominantly implicated, such as adipocytes in obesity and diabetes and kidney cells in chronic kidney disease (Figure 1). Accordingly, it has been proposed that ANGPTL2 does not exert systemic effects on distant organs and that it rather acts locally: in a transgenic mouse model of ANGPTL2 overexpression in the skin (K14-ANGPTL2-Tg mice), renal fibrosis was not observed after unilateral uretal obstruction despite higher systemic ANGPTL2 levels [49], suggesting that it is the kidney-derived ANGPTL2 that causes renal fibrosis, not a global increase in systemic ANGPTL2 levels. In addition, one study showed that cardiomyocyte inactivation of ANGPTL2 synthesis using AAV6-shAngptl2 injection following heart failure in mice reduced circulating levels of ANGPTL2 and improved cardiac function [68], and recent data suggest that ANGPTL2 secreted by HSC in the circulation may exert local roles in its microenvironment to maintain stemness [83], supporting indeed that cell-specific production of ANGPTL2 may be associated with specific organ defects/function. This hypothesis needs however to be substantiated by more studies. Elucidation of what type of cell expresses ANGPTL2, its receptor(s) and how receptor-mediated signaling of ANGPTL2 is regulated in target cells will therefore enable deeper understanding of the role of ANGPTL2 in the pathogenesis of different diseases and the physiological roles of ANGPTL2 such as maintenance of stemness.

In conclusion, the clinical prognostic value of abnormal ANGPTL2 circulating levels should be confirmed and ANGPTL2 could then be included in the routine blood biochemical marker panel in association with the inflammatory marker hsCRP, the cardiac marker NT-proBNP or tumour markers such as carcinoembryonic antigen (CEA) and carbohydrate antigen19-9 (CA19-9). It is important to note, however, that the upper threshold for normal serum ANGPTL2 concentrations has not been clearly defined. A range between 1 and 3 ng/ml is usually considered normal, in healthy physically active controls, and when circulating levels of ANGPTL2 rise chronically, even slightly, this is associated with the development and progression of a chronic disease. When secreted by adipocytes, endothelial cells, macrophages, cardiomyocytes, kidney cells, hematopoietic stem cells, or cancer cells, ANGPTL2 acts rather locally, exhibiting both autocrine and paracrine effects, leading to physiological and pathological effects. We believe that elevated circulating levels of ANGPTL2 are not a biomarker of a specific disease but may rather reflect a cellular dysfunction pathway and/or the senescent cellular load of an individual.

## Figures and Tables

**Table 1:** Circulating levels of ANGPTL2 in cardiovascular diseases.

Reference	Patients	Serum ANGPTL2 (ng/ml)	ELISA kit	Conclusion of the study
[48]	Chronic kidney disease (stage 5) ± kidney transplant, Canada	from 71 [53–95] to 11 [9–15] after kidney transplant	USCN Life Science Inc., China	High ANGPTL2 after kidney transplant is associated with aortic stiffness, pulse pressure, renal function, and mortality.
[44]	Hisayama study, general population, Japan	Q1: <2.2 Q4: >3.4	IBL, Japan	High ANGPTL2 is an independent factor for T2DM development.
[50]	Coronary artery disease versus age-matched controls, Canada	~6.0 versus 1.0 in controls	USCN Life Science Inc., China	ANGPTL2 promotes atherosclerosis in mice. ANGPTL2 is higher in CAD patients.
[45]	Diabetes and chronic kidney disease (<stage 4), France	Q1: <11.2 Q4: >19.5	USCN Life Science Inc., China	ANGPTL2 improves risk stratification inT2DM: Q4 predicts MACE and mortality.
[51]	Hisayama study, general Japanese population	Q1: <2.25 Q4: >3.62	IBL, Japan	High ANGPTL2 is an independent factor for cardiovascular disease development.
[26]	Coronary heart disease versus controls, Japan	~4.6 versus 3.6 in controls	IBL, Japan	ANGPTL2 is higher in coronary heart disease patients.
	Seniors (85–99 years old), general population, Japan	4.1 [3.2–5.1]		ANGPTL2 correlates with inflammation, IMT, and the presence of plaque: ANGPTL2 reflects atherosclerosis.
[52]	Heart failure versus age-matched controls, Taiwan	~4.6 versus 3.5 in controls T1: <3.4 T3: >4.8	IBL, Japan	ANGPTL2 is higher in HF patients. Higher risk (2.97-fold) of HF in patients with ANGPTL2 levels in T3.
[53]	T2DM, South Korea	Q1: <3.3 Q4: >5.2	IBL, Japan	ANGPTL2 correlates with carotid IMT: ANGPTL2 is important in atherosclerosis.
[7]	Coronary artery disease versus age-matched controls, Canada	~5.5 versus 2.0 in controls	USCN Life Science Inc., China	ANGPTL2 is higher in CAD patients. Acute intermittent exercise reduces ANGPTL2.
[46]	Diabetes with nephropathy versus controls, China	36.1 to 50.2 versus 24.0 in controls	USCN Life Science Inc., China	ANGPTL2 is independently associated with albuminuria: role of ANGPTL2 in nephropathy in T2DM patients.
[58]	Obese metabolically healthy women China	4.2 (at risk for insulin resistance) versus 2.9 (normal IS)	Not mentioned	ANGPTL2 is negatively correlated with insulin sensitivity and serum epinephrine levels.
[59]	Overweight healthy men, Japan	~3.0 versus 2.8 after diet and exercise	IBL, Japan	Lifestyle intervention reduces ANGPTL2. Changes in ANGPTL2 reflect visceral fat and metabolic improvement.
[54]	Acute coronary syndrome versus age-matched controls, Canada	~3.4 versus 1.8 in controls	USCN Life Science Inc., China	ANGPTL2 is higher in acute coronary syndrome patients and is associated with reduced leukocyte DNA methylation in the promoter region of *ANGPTL2* gene.
[55]	Coronary artery disease versus controls, Japan	~4.0–6.7 versus 3.0 in controls	IBL, Japan	ANGPTL2 is higher in patients with multivessel CAD than in those with single vessel disease.
[60]	Severe obese ± bariatric surgery, Canada	12.3 (9.3–14.9)	USCN Life Science Inc., China	Bariatric surgery decreases ANGPTL2 and this is associated with a better cardiometabolic profile, not with anthropometric parameters.
[77]	Patients with acute myocardial infarction, Japan	~2.0	IBL, Japan	Statin started early after the onset of myocardial infarction reduces ANGPTL2.
[5]	Coronary artery disease, obese, diabetes versus controls, Japan	~4.0–5.0 versus 2.5 in controls	IBL, Japan	ANGPTL2 is a key adipocyte-derived inflammatory mediator that links obesity to insulin resistance.
[8]	Post-acute coronary syndrome patients, effect of exercise, Canada	Men: from 2.8 to 1.4 after EX Women: from 4.4 to 5.1 after EX	USCN Life Science Inc., China	In post ACS men (not women), ANGPTL2 is reduced by exercise training. ANGPTL2 reached at the end of the training reflects endothelial and cardiopulmonary functions.
[68]	Dilated cardiomyopathy, Japan	Coronary sinus: 4.6 versus 2.1 Aortic root: 2.4 versus 2.5	IBL, Japan	A difference between ANGPTL2 in coronary sinus and aortic root reflects ANGPTL2 secretion from the heart.
[9]	Hisayama study, general population, Japan	Q1: 2.0 Q4: >3.7	IBL, Japan	High ANGPTL2 is associated with the prevalence of chronic kidney disease.
[57]	Acute coronary syndrome versus controls, China	Q1: <11.3 Q4: >43.7 versus 7–25 in controls	Cusabio, China	ANGPTL2 is closely associated with ACS and provides risk stratification of the disease.
[47]	Gestational diabetes, China	Q1: <2.0 Q4: >3.5	IBL, Japan	ANGPTL2 is higher in women with gestational diabetes. The risk of developing gestational diabetes is x2.9 in Q4.

ACS: acute coronary syndrome; CAD: coronary artery disease; EX: exercise; HF: heart failure; IMT: intima-media thickness; IS: insulin sensitivity; MACE: major adverse cardiovascular events; Q: quartile; T2DM: type 2 diabetes mellitus; T: tertile.

**Table 2:** Circulating levels of ANGPTL2 in cancer.

Reference	Patients	Serum ANGPTL2 (ng/ml)	ELISA kit	Conclusion of the study
[30]	Non-small-cell lung cancer, China	8.4 ± 1.7 versus 4.9 ± 1.0 in controls	USCN Life Science Inc., China	High ANGPTL2 is a novel potential biomarker for diagnosis and prognosis of patients with non-small-cell lung cancer.
[32]	Breast cancer, Japan	~4.0 versus 2.0 in controls	IBL, Japan	High ANGPTL2 in breast cancer patients could represent a potential marker of breast cancer metastasis.
[33]	Hepatocellular carcinoma, China	Values not given (ANGPTL2 not increased in cancer patients)	Not mentioned	ANGPTL2 drives hepatocellular carcinoma metastasis.
[34]	Esophageal cancer, Japan	~1.5 versus 0.8 in controls	IBL, Japan	High ANGPTL2 is a novel biomarker for diagnosis and prognosis of patients with esophageal cancer.
[40]	Colorectal cancer, Japan	~1.5 versus 0.8 in controls	IBL, Japan	ANGPTL2 is a potential marker for diagnosis, early recurrence and prognosis in colorectal cancer patients.
[38]	Colorectal cancer, Japan	1.9 (meta (+)) versus 1.5 (meta (−))	IBL, Japan	ANGPTL2 improves preoperative detection of lymph node metastasis in colorectal cancer.
[39]	Gastric cancer, Japan	~1.5 versus 0.8 in controls	IBL, Japan	High ANGPTL2 correlates with the metastatic properties of gastric cancer and could be a biomarker for early diagnosis and recurrence.
[41]	Colorectal cancer, Japan	~3.5 versus 2.7 in controls	IBL, Japan	High ANGPTL2 could be a potential biomarker for early detection of colorectal cancer.
[42]	Gastric cancer, Japan	~3.6 versus 2.7 in controls	IBL, Japan	High ANGPTL2 could be a potential biomarker for gastric cancer.

**Table 3:** Circulating levels of ANGPTL2 in autoimmune diseases.

Reference	Patients	Serum ANGPTL2 (ng/ml)	ELISA kit	Conclusion of the study
[61]	Dermatomyositis versus controls, Japan	~3.8 versus 3.0 in controls	IBL, Japan	Keratinocyte-derived ANGPTL2 contributes to DM pathogenesis by inducing chronic inflammation in skin tissue.
[62]	Rheumatoid arthritis versus controls, Japan	~3.0 versus 3.0 in controls (NS)	IBL, Japan	Synovial fluid-derived ANGPTL2 (not serum) acts as an important rheumatoid inflammatory mediator in RA pathogenesis.

DM: dermatomyositis; NS: nonsignificant; RA: rheumatoid arthritis.
